# 3-Methyl-5-(4-methyl­phen­yl)cyclo­hex-2-enone

**DOI:** 10.1107/S1600536808013822

**Published:** 2008-05-14

**Authors:** R. T. Sabapathy Mohan, S. Kamatchi, M. Subramanyam, A. Thiruvalluvar, A. Linden

**Affiliations:** a Department of Chemistry, Annamalai University, Annamalai Nagar 608 002, Tamil Nadu, India; b PG Research Department of Physics, Rajah Serfoji Government College (Autonomous), Thanjavur 613 005, Tamil Nadu, India; c Institute of Organic Chemistry, University of Zürich, Winterthurerstrasse 190, CH-8057 Zürich, Switzerland.

## Abstract

In the title mol­ecule, C_14_H_16_O, the cyclo­hexene ring adopts an envelope conformation, with all substituents equatorial. Mol­ecules are linked by C—H⋯O hydrogen bonds. A C—H⋯π inter­action involving the benzene ring is also found in the crystal structure. The H atoms of both methyl groups are disordered equally over two positions.

## Related literature

For related literature, see: Padmavathi *et al.* (2000 ▶).
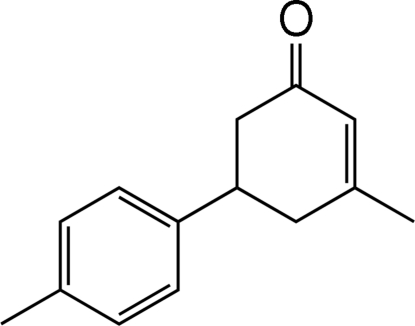


## Experimental

### 

#### Crystal data

C_14_H_16_O
*M* *_r_* = 200.27
Monoclinic, 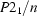

*a* = 5.2623 (3) Å
*b* = 11.1583 (7) Å
*c* = 19.3341 (11) Å
β = 94.994 (4)°
*V* = 1130.96 (12) Å^3^
*Z* = 4
Mo *K*α radiation
μ = 0.07 mm^−1^
*T* = 160 (1) K
0.25 × 0.18 × 0.18 mm

#### Data collection

Nonius KappaCCD area-detector diffractometer
Absorption correction: none
16716 measured reflections
2002 independent reflections
1316 reflections with *I* > 2σ(*I*)
*R*_int_ = 0.085

#### Refinement

*R*[*F* ^2^ > 2σ(*F* ^2^)] = 0.072
*wR*(*F* ^2^) = 0.231
*S* = 1.09
2002 reflections
137 parameters
H-atom parameters constrained
Δρ_max_ = 0.48 e Å^−3^
Δρ_min_ = −0.30 e Å^−3^

### 

Data collection: *COLLECT* (Nonius, 2000 ▶); cell refinement: *DENZO-SMN* (Otwinowski & Minor, 1997 ▶); data reduction: *DENZO-SMN* and *SCALEPACK* (Otwinowski & Minor, 1997 ▶); program(s) used to solve structure: *SHELXS97* (Sheldrick, 2008 ▶); program(s) used to refine structure: *SHELXL97* (Sheldrick, 2008 ▶); molecular graphics: *ORTEP-3* (Farrugia, 1997 ▶); software used to prepare material for publication: *PLATON* (Spek, 2003 ▶).

## Supplementary Material

Crystal structure: contains datablocks global, I. DOI: 10.1107/S1600536808013822/wn2260sup1.cif

Structure factors: contains datablocks I. DOI: 10.1107/S1600536808013822/wn2260Isup2.hkl

Additional supplementary materials:  crystallographic information; 3D view; checkCIF report

## Figures and Tables

**Table 1:** Hydrogen-bond geometry (Å, °)

*D*—H⋯*A*	*D*—H	H⋯*A*	*D*⋯*A*	*D*—H⋯*A*
C2—H2⋯O1^i^	0.95	2.48	3.425 (3)	173
C5—H5⋯*Cg*^ii^	1.00	2.94	3.818 (3)	147

Symmetry codes: (i) 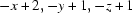; (ii) 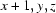. *Cg* is the centroid of the bezene ring.

**Fig. 1.**
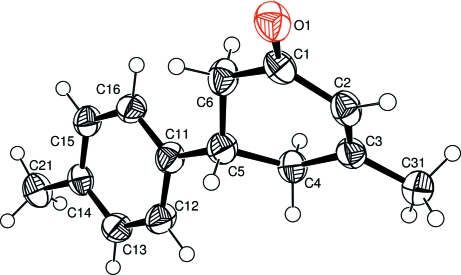
The molecular structure of the title compound, showing the atom-numbering scheme and displacement ellipsoids drawn at the 50% probability level. Hydrogen atoms are represented by spheres of arbitrary radius.

**Fig. 2.**
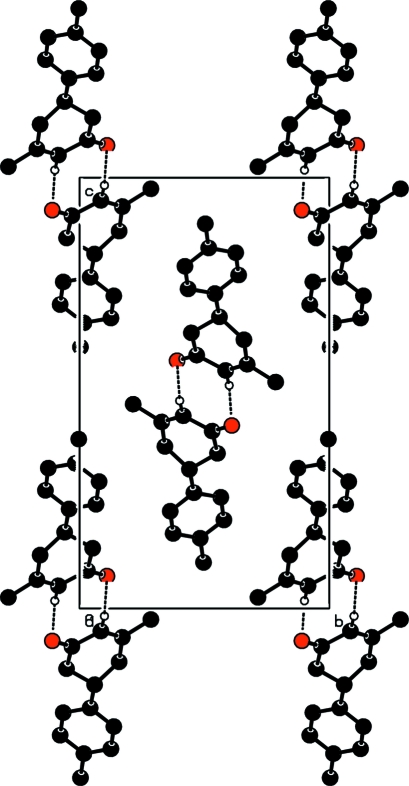
The molecular packing of the title compound, viewed down the a axis showing the C—H···O (dashed lines) interactions.

## supplementary crystallographic information

### Comment

The title compound, has been analysed as part of our crystallographic studies on substituted cyclohexenes. The molecular structure of the title compound, with atomic numbering scheme, is shown in Fig. 1. The cyclohexene ring adopts an envelope conformation, with all substituents equatorial. Molecules are linked by C2—H2···O1(2 - *x*, 1 - *y*, 1 - *z*) hydrogen bonds (Fig. 2). A C—H···π interaction involving the benzene ring is also found in the crystal structure.

### Experimental

The title compound was prepared according to the general procedure reported by Padmavathi *et al.* (2000). A mixture of 2,4-bis(ethoxycarbonyl)-5-hydroxy-5-methyl-3,4'-methylphenylcyclohexanone (3.62 g, 0.01 mol) in glacial acetic acid (25 ml) and concentrated hydrochloric acid (50 ml) was refluxed for 12 h. After completion of the reaction, the reaction mixture was neutralized with aqueous ammonia and separated using chloroform. The product was purified by column chromatography (benzene-EtOAc, 9.5:0.5 *v*/*v*). The yield of the isolated product was 1.07 g (87%).

### Refinement

H atoms were positioned geometrically and allowed to ride on their parent atoms, with C—H = 0.95–1.00 Å and *U*_iso_(H) = *xU*_eq_(carrier atom), where *x* = 1.5 for methyl and 1.2 for all other C atoms. The H atoms of both methyl groups were found to be disordered equally over two positions rotated from each other by 60°. They were refined as idealized.

### Crystal data

**Table:**

C_14_H_16_O	*F*_000_ = 432
*M**_r_* = 200.27	*D*_x_ = 1.176 Mg m^−^^3^
Monoclinic, *P*2_1_/*n*	Melting point: 315 K
Hall symbol: -P 2yn	Mo *K*α radiation λ = 0.71073 Å
*a* = 5.2623 (3) Å	Cell parameters from 2105 reflections
*b* = 11.1583 (7) Å	θ = 2.0–25.0º
*c* = 19.3341 (11) Å	µ = 0.07 mm^−^^1^
β = 94.994 (4)º	*T* = 160 (1) K
*V* = 1130.96 (12) Å^3^	Prism, colourless
*Z* = 4	0.25 × 0.18 × 0.18 mm

### Data collection

**Table:**

Nonius KappaCCD area-detector diffractometer	2002 independent reflections
Radiation source: Nonius FR590 sealed tube generator	1316 reflections with *I* > 2σ(*I*)
Monochromator: horizontally mounted graphite crystal	*R*_int_ = 0.085
Detector resolution: 9 pixels mm^-1^	θ_max_ = 25.0º
*T* = 160(1) K	θ_min_ = 3.7º
ω scans with κ offsets	*h* = 0→6
Absorption correction: none	*k* = 0→13
16716 measured reflections	*l* = −22→22

### Refinement

**Table:**

Refinement on *F*^2^	Hydrogen site location: inferred from neighbouring sites
Least-squares matrix: full	H-atom parameters constrained
*R*[*F*^2^ > 2σ(*F*^2^)] = 0.072	*w* = 1/[σ^2^(*F*_o_^2^) + (0.1423*P*)^2^] where *P* = (*F*_o_^2^ + 2*F*_c_^2^)/3
*wR*(*F*^2^) = 0.231	(Δ/σ)_max_ < 0.001
*S* = 1.09	Δρ_max_ = 0.48 e Å^−^^3^
2002 reflections	Δρ_min_ = −0.30 e Å^−^^3^
137 parameters	Extinction correction: SHELXL97 (Sheldrick, 2008), Fc^*^=kFc[1+0.001xFc^2^λ^3^/sin(2θ)]^-1/4^
Primary atom site location: structure-invariant direct methods	Extinction coefficient: 0.16 (2)
Secondary atom site location: difference Fourier map	

### Special details

**Table:**

Experimental. Solvent used: ? Cooling Device: Oxford Cryosystems Cryostream 700 Crystal mount: glued on a glass fibre Mosaicity (°.): 0.728 (3) Frames collected: 237 Seconds exposure per frame: 18 Degrees rotation per frame: 1.8 Crystal-Detector distance (mm): 30.0
Geometry. Bond distances, angles *etc*. have been calculated using the rounded fractional coordinates. All su's are estimated from the variances of the (full) variance-covariance matrix. The cell e.s.d.'s are taken into account in the estimation of distances, angles and torsion angles
Refinement. Refinement of *F*^2^ against ALL reflections. The weighted *R*-factor *wR* and goodness of fit *S* are based on *F*^2^, conventional *R*-factors *R* are based on *F*, with *F* set to zero for negative *F*^2^. The threshold expression of *F*^2^ > σ(*F*^2^) is used only for calculating *R*-factors(gt) *etc*. and is not relevant to the choice of reflections for refinement. *R*-factors based on *F*^2^ are statistically about twice as large as those based on *F*, and *R*- factors based on ALL data will be even larger.

### Fractional atomic coordinates and isotropic or equivalent isotropic displacement parameters (Å^2^)

**Table:**

	*x*	*y*	*z*	*U*_iso_*/*U*_eq_	Occ. (<1)
O1	0.8289 (3)	0.60964 (17)	0.42477 (10)	0.0480 (7)	
C1	0.6687 (5)	0.5310 (2)	0.41187 (13)	0.0380 (9)	
C2	0.6807 (5)	0.4150 (3)	0.44740 (14)	0.0392 (9)	
C3	0.5102 (5)	0.3288 (2)	0.43313 (13)	0.0373 (9)	
C4	0.2974 (5)	0.3417 (2)	0.37608 (13)	0.0397 (9)	
C5	0.3519 (5)	0.4399 (2)	0.32304 (14)	0.0410 (9)	
C6	0.4432 (5)	0.5523 (3)	0.36013 (15)	0.0446 (10)	
C11	0.1274 (5)	0.4579 (2)	0.26887 (14)	0.0399 (9)	
C12	0.0467 (5)	0.3630 (3)	0.22587 (15)	0.0449 (10)	
C13	−0.1517 (5)	0.3746 (3)	0.17411 (15)	0.0437 (10)	
C14	−0.2758 (5)	0.4829 (2)	0.16303 (13)	0.0386 (9)	
C15	−0.1973 (5)	0.5781 (2)	0.20557 (14)	0.0412 (9)	
C16	−0.0006 (5)	0.5656 (2)	0.25810 (14)	0.0411 (9)	
C21	−0.4928 (5)	0.4965 (3)	0.10693 (15)	0.0505 (10)	
C31	0.5112 (6)	0.2145 (2)	0.47430 (15)	0.0496 (10)	
H2	0.81624	0.40075	0.48216	0.0471*	
H4A	0.27208	0.26420	0.35156	0.0476*	
H4B	0.13730	0.36148	0.39696	0.0476*	
H5	0.49701	0.41021	0.29761	0.0492*	
H6A	0.49029	0.61187	0.32552	0.0536*	
H6B	0.30195	0.58644	0.38449	0.0536*	
H12	0.13007	0.28779	0.23210	0.0539*	
H13	−0.20263	0.30744	0.14604	0.0525*	
H15	−0.27932	0.65353	0.19880	0.0494*	
H16	0.04671	0.63215	0.28705	0.0493*	
H21A	−0.55656	0.57899	0.10675	0.0758*	0.500
H21B	−0.43159	0.47801	0.06168	0.0758*	0.500
H21C	−0.63066	0.44117	0.11600	0.0758*	0.500
H21D	−0.52265	0.41979	0.08287	0.0758*	0.500
H21E	−0.64762	0.52077	0.12794	0.0758*	0.500
H21F	−0.44855	0.55761	0.07362	0.0758*	0.500
H31A	0.37072	0.16309	0.45556	0.0744*	0.500
H31B	0.67370	0.17279	0.47117	0.0744*	0.500
H31C	0.48990	0.23312	0.52299	0.0744*	0.500
H31D	0.65216	0.21624	0.51092	0.0744*	0.500
H31E	0.34918	0.20654	0.49531	0.0744*	0.500
H31F	0.53298	0.14621	0.44349	0.0744*	0.500

### Atomic displacement parameters (Å^2^)

**Table:**

	*U*^11^	*U*^22^	*U*^33^	*U*^12^	*U*^13^	*U*^23^
O1	0.0439 (12)	0.0548 (13)	0.0434 (13)	−0.0079 (10)	−0.0074 (9)	0.0016 (9)
C1	0.0331 (15)	0.0480 (17)	0.0324 (15)	−0.0016 (13)	0.0006 (12)	−0.0033 (12)
C2	0.0340 (14)	0.0505 (17)	0.0324 (15)	0.0048 (13)	−0.0014 (12)	0.0012 (12)
C3	0.0368 (15)	0.0427 (16)	0.0323 (15)	0.0072 (12)	0.0025 (12)	−0.0009 (12)
C4	0.0379 (16)	0.0433 (17)	0.0370 (16)	0.0007 (12)	−0.0012 (12)	0.0020 (12)
C5	0.0421 (16)	0.0400 (16)	0.0393 (16)	0.0006 (12)	−0.0052 (13)	0.0000 (12)
C6	0.0429 (16)	0.0458 (17)	0.0432 (17)	−0.0035 (13)	−0.0076 (13)	0.0081 (13)
C11	0.0393 (16)	0.0390 (16)	0.0399 (16)	−0.0028 (12)	−0.0043 (12)	0.0024 (12)
C12	0.0485 (17)	0.0373 (16)	0.0463 (18)	0.0028 (12)	−0.0114 (14)	−0.0005 (12)
C13	0.0471 (17)	0.0418 (17)	0.0406 (17)	−0.0031 (13)	−0.0058 (13)	−0.0043 (12)
C14	0.0366 (15)	0.0468 (17)	0.0315 (15)	−0.0018 (12)	−0.0024 (11)	0.0029 (12)
C15	0.0403 (16)	0.0424 (16)	0.0395 (17)	0.0052 (12)	−0.0049 (13)	0.0046 (12)
C16	0.0449 (16)	0.0360 (16)	0.0409 (17)	−0.0035 (12)	−0.0042 (13)	−0.0015 (12)
C21	0.0443 (17)	0.065 (2)	0.0398 (17)	0.0002 (15)	−0.0103 (13)	0.0021 (14)
C31	0.0583 (19)	0.0449 (17)	0.0441 (17)	0.0061 (14)	−0.0038 (14)	0.0024 (13)

### Geometric parameters (Å, °)

**Table:**

O1—C1	1.227 (3)	C5—H5	1.0000
C1—C2	1.464 (4)	C6—H6A	0.9900
C1—C6	1.503 (4)	C6—H6B	0.9900
C2—C3	1.328 (4)	C12—H12	0.9500
C3—C4	1.509 (4)	C13—H13	0.9500
C3—C31	1.503 (3)	C15—H15	0.9500
C4—C5	1.545 (3)	C16—H16	0.9500
C5—C6	1.503 (4)	C21—H21A	0.9800
C5—C11	1.522 (4)	C21—H21B	0.9800
C11—C12	1.390 (4)	C21—H21C	0.9800
C11—C16	1.385 (3)	C21—H21D	0.9800
C12—C13	1.388 (4)	C21—H21E	0.9800
C13—C14	1.381 (4)	C21—H21F	0.9800
C14—C15	1.384 (3)	C31—H31A	0.9800
C14—C21	1.513 (4)	C31—H31B	0.9800
C15—C16	1.393 (4)	C31—H31C	0.9800
C2—H2	0.9500	C31—H31D	0.9800
C4—H4A	0.9900	C31—H31E	0.9800
C4—H4B	0.9900	C31—H31F	0.9800
			
O1···C31^i^	3.387 (3)	H4B···H2^v^	2.5000
O1···H6B^ii^	2.6800	H5···C2	2.9700
O1···H13^iii^	2.6500	H5···C14^ii^	3.0700
O1···H2^iv^	2.4800	H6A···C15^ii^	2.9800
O1···H31C^i^	2.6800	H6A···C16	2.8400
O1···H31E^i^	2.7800	H6A···H16	2.4000
C1···C16^ii^	3.594 (4)	H6A···C12^iii^	2.9700
C1···C2^i^	3.467 (4)	H6A···C13^iii^	3.0500
C1···C3^i^	3.579 (4)	H6A···H12^iii^	2.3200
C2···C1^i^	3.467 (4)	H6A···H13^iii^	2.4900
C2···C2^i^	3.468 (4)	H6B···O1^v^	2.6800
C3···C1^i^	3.579 (4)	H6B···C16	2.8100
C16···C1^v^	3.594 (4)	H6B···H16	2.2700
C31···O1^i^	3.387 (3)	H12···C4	2.9100
C1···H31C^i^	3.0600	H12···H4A	2.3800
C2···H5	2.9700	H12···H6A^vi^	2.3200
C2···H4B^ii^	2.7400	H12···H16^vi^	2.4800
C4···H12	2.9100	H13···H21D	2.3500
C6···H16	2.5700	H13···O1^vi^	2.6500
C12···H21C^ii^	2.9600	H13···H6A^vi^	2.4900
C12···H4A	2.8300	H15···H21A	2.3500
C12···H6A^vi^	2.9700	H16···C6	2.5700
C13···H6A^vi^	3.0500	H16···H6A	2.4000
C14···H5^v^	3.0700	H16···H6B	2.2700
C15···H6A^v^	2.9800	H16···H12^iii^	2.4800
C16···H6A	2.8400	H21A···H15	2.3500
C16···H6B	2.8100	H21A···H4A^vii^	2.5200
C21···H31B^iii^	3.0700	H21C···C12^v^	2.9600
C21···H31A^vii^	2.9100	H21D···H13	2.3500
C31···H21F^vi^	3.1000	H21F···C31^iii^	3.1000
H2···H4B^ii^	2.5000	H31A···H4A	2.3200
H2···H31D	2.3200	H31A···C21^viii^	2.9100
H2···O1^iv^	2.4800	H31B···C21^vi^	3.0700
H4A···C12	2.8300	H31C···O1^i^	2.6800
H4A···H12	2.3800	H31C···C1^i^	3.0600
H4A···H31A	2.3200	H31D···H2	2.3200
H4A···H31F	2.5200	H31E···O1^i^	2.7800
H4A···H21A^viii^	2.5200	H31F···H4A	2.5200
H4B···C2^v^	2.7400		
			
O1—C1—C2	122.4 (2)	C15—C16—H16	119.00
O1—C1—C6	120.7 (2)	C14—C21—H21A	109.00
C2—C1—C6	116.8 (2)	C14—C21—H21B	109.00
C1—C2—C3	122.8 (2)	C14—C21—H21C	109.00
C2—C3—C4	121.9 (2)	C14—C21—H21D	109.00
C2—C3—C31	122.2 (2)	C14—C21—H21E	109.00
C4—C3—C31	115.9 (2)	C14—C21—H21F	109.00
C3—C4—C5	112.6 (2)	H21A—C21—H21B	109.00
C4—C5—C6	110.2 (2)	H21A—C21—H21C	110.00
C4—C5—C11	111.9 (2)	H21A—C21—H21D	141.00
C6—C5—C11	114.5 (2)	H21A—C21—H21E	56.00
C1—C6—C5	112.8 (2)	H21A—C21—H21F	56.00
C5—C11—C12	119.3 (2)	H21B—C21—H21C	109.00
C5—C11—C16	123.8 (2)	H21B—C21—H21D	56.00
C12—C11—C16	116.9 (2)	H21B—C21—H21E	141.00
C11—C12—C13	122.1 (3)	H21B—C21—H21F	56.00
C12—C13—C14	120.6 (3)	H21C—C21—H21D	56.00
C13—C14—C15	117.8 (2)	H21C—C21—H21E	56.00
C13—C14—C21	121.1 (2)	H21C—C21—H21F	141.00
C15—C14—C21	121.1 (2)	H21D—C21—H21E	109.00
C14—C15—C16	121.4 (2)	H21D—C21—H21F	109.00
C11—C16—C15	121.2 (2)	H21E—C21—H21F	110.00
C1—C2—H2	119.00	C3—C31—H31A	109.00
C3—C2—H2	119.00	C3—C31—H31B	109.00
C3—C4—H4A	109.00	C3—C31—H31C	109.00
C3—C4—H4B	109.00	C3—C31—H31D	109.00
C5—C4—H4A	109.00	C3—C31—H31E	109.00
C5—C4—H4B	109.00	C3—C31—H31F	109.00
H4A—C4—H4B	108.00	H31A—C31—H31B	109.00
C4—C5—H5	107.00	H31A—C31—H31C	109.00
C6—C5—H5	107.00	H31A—C31—H31D	141.00
C11—C5—H5	107.00	H31A—C31—H31E	56.00
C1—C6—H6A	109.00	H31A—C31—H31F	56.00
C1—C6—H6B	109.00	H31B—C31—H31C	109.00
C5—C6—H6A	109.00	H31B—C31—H31D	56.00
C5—C6—H6B	109.00	H31B—C31—H31E	141.00
H6A—C6—H6B	108.00	H31B—C31—H31F	56.00
C11—C12—H12	119.00	H31C—C31—H31D	56.00
C13—C12—H12	119.00	H31C—C31—H31E	56.00
C12—C13—H13	120.00	H31C—C31—H31F	141.00
C14—C13—H13	120.00	H31D—C31—H31E	109.00
C14—C15—H15	119.00	H31D—C31—H31F	109.00
C16—C15—H15	119.00	H31E—C31—H31F	109.00
C11—C16—H16	119.00		
			
O1—C1—C2—C3	−179.1 (3)	C4—C5—C11—C16	120.6 (3)
C6—C1—C2—C3	3.3 (4)	C6—C5—C11—C12	172.5 (2)
O1—C1—C6—C5	149.3 (2)	C6—C5—C11—C16	−5.7 (4)
C2—C1—C6—C5	−33.1 (3)	C5—C11—C12—C13	−178.0 (3)
C1—C2—C3—C4	3.3 (4)	C16—C11—C12—C13	0.4 (4)
C1—C2—C3—C31	−174.4 (2)	C5—C11—C16—C15	177.0 (2)
C2—C3—C4—C5	19.3 (3)	C12—C11—C16—C15	−1.3 (4)
C31—C3—C4—C5	−162.8 (2)	C11—C12—C13—C14	0.6 (4)
C3—C4—C5—C6	−47.3 (3)	C12—C13—C14—C15	−0.7 (4)
C3—C4—C5—C11	−175.9 (2)	C12—C13—C14—C21	−179.8 (3)
C4—C5—C6—C1	54.2 (3)	C13—C14—C15—C16	−0.3 (4)
C11—C5—C6—C1	−178.6 (2)	C21—C14—C15—C16	178.9 (2)
C4—C5—C11—C12	−61.2 (3)	C14—C15—C16—C11	1.3 (4)

Symmetry codes: (i) −*x*+1, −*y*+1, −*z*+1; (ii) *x*+1, *y*, *z*; (iii) −*x*+1/2, *y*+1/2, −*z*+1/2; (iv) −*x*+2, −*y*+1, −*z*+1; (v) *x*−1, *y*, *z*; (vi) −*x*+1/2, *y*−1/2, −*z*+1/2; (vii) −*x*−1/2, *y*+1/2, −*z*+1/2; (viii) −*x*−1/2, *y*−1/2, −*z*+1/2.

### Hydrogen-bond geometry (Å, °)

**Table:**

*D*—H···*A*	*D*—H	H···*A*	*D*···*A*	*D*—H···*A*
C2—H2···O1^iv^	0.95	2.48	3.425 (3)	173
C5—H5···Cg^ii^	1.00	2.94	3.818 (3)	147

Symmetry codes: (iv) −*x*+2, −*y*+1, −*z*+1; (ii) *x*+1, *y*, *z*.

## References

[bb1] Farrugia, L. J. (1997). *J. Appl. Cryst.* **30**, 565.

[bb2] Nonius (2000). *COLLECT* Nonius BV, Delft, The Netherlands.

[bb3] Otwinowski, Z. & Minor, W. (1997). *Methods in Enzymology*, Vol. 276, *Macromolecular Crystallography*, Part A, edited by C. W. Carter Jr & R. M. Sweet, pp. 307–326. London: Academic Press.

[bb4] Padmavathi, V., Jagan Mohan Reddy, B., Balaih, A., Venugopal Reddy, K. & Bhasker Reddy, D. (2000). *Molecules.* **5**, 1281–1286.

[bb5] Sheldrick, G. M. (2008). *Acta Cryst.* A**64**, 112–122. 10.1107/S0108767307043930 18156677

[bb6] Spek, A. L. (2003). *J. Appl. Cryst.* **36**, 7–13.

